# Stability and Photoisomerization of Stilbenes Isolated from the Bark of Norway Spruce Roots

**DOI:** 10.3390/molecules26041036

**Published:** 2021-02-16

**Authors:** Harri Latva-Mäenpää, Riziwanguli Wufu, Daniel Mulat, Tytti Sarjala, Pekka Saranpää, Kristiina Wähälä

**Affiliations:** 1 Department of Chemistry, University of Helsinki, P.O. Box 55, FI-00014 Helsinki, Finland; riziwanguli.wufu@helsinki.fi (R.W.); d.mulat@cgiar.org (D.M.); 2 Foodwest, Kärryväylä 4, FI-60100 Seinäjoki, Finland; 3 Natural Resources Institute Finland, Tietotie 2, FI-02150 Espoo, Finland; tytti.sarjala@luke.fi; 4 Department of Biochemistry and Developmental Biology, University of Helsinki, P.O. Box 63, FI-00014 Helsinki, Finland

**Keywords:** stilbenes, spruce bark, UV-light, fluorescent light, photoisomerization, *trans* to *cis* isomerization, phenanthrenes, astringin, piceid, isorhapontin, piceatannol, isorhapontigenin

## Abstract

Stilbenes or stilbenoids, major polyphenolic compounds of the bark of Norway spruce (*Picea abies* L. Karst), have potential future applications as drugs, preservatives and other functional ingredients due to their antioxidative, antibacterial and antifungal properties. Stilbenes are photosensitive and UV and fluorescent light induce *trans* to *cis* isomerisation via intramolecular cyclization. So far, the characterizations of possible new compounds derived from *trans*-stilbenes under UV light exposure have been mainly tentative based only on UV or MS spectra without utilizing more detailed structural spectroscopy techniques such as NMR. The objective of this work was to study the stability of biologically interesting and readily available stilbenes such as astringin and isorhapontin and their aglucones piceatannol and isorhapontigenin, which have not been studied previously. The effects of fluorescent and UV light and storage on the stability of *trans* stilbenes were assessed and the identification and characterisation of new compounds formed during our experiments were carried out by chromatographic (HPLC, GC) and spectroscopic techniques (UV, MS, NMR). The stilbenes undergo a *trans* to *cis* isomerisation under extended UV irradiation by intramolecular cyclisation (by the formation of a new C-C bond and the loss of two hydrogens) to phenanthrene structures. The characterised compounds are novel and not described previously.

## 1. Introduction

Stilbenes or stilbenoids are expected to show many future applications as drugs and functional ingredients or preservatives in food products or improving material properties. The best known stilbenoid compound is resveratrol, but there are also other interesting stilbenoids such as astringin and isorhapontin, which are derived from forest biomass and are potential starting materials for new products. The bark of Norway spruce (*Picea abies* L. Karst) contains the stilbenoid glucosides astringin and isorhapontin as the major polyphenolic compounds that are even found in roots [1]. Stilbenes are secondary metabolites and play an important role in the tree defence mechanism [2]. They provide protection against ultraviolet (UV) light and attacks by fungal pathogens and other microorganisms [3,4,5] and they also show potential to prevent pathologies associated with oxidative stress [6]. Stilbenoid glucosides are mainly localized to the inner part of the bark in the functional phloem [7] and they can be converted to their aglucones by enzymatic hydrolysis [8].

Stilbenes may exist as the *trans* and *cis* stereoisomeric forms, but naturally they overwhelmingly occur as the *trans* isomers due to their higher thermodynamical stability compared to the *cis* isomers [9]. However, *trans*-stilbenes may be isomerized to the *cis* form under certain conditions such as irradiation with light. Recently, Välimaa and co-authors [10] showed that UVA-induced modification of the stilbene-rich inner bark extracts increased their radical scavenging activity. The results even indicated a slightly increased antimicrobial activity after UVA-modification. The authors concluded that stilbenes from spruce bark may have potential as preservatives.

The conflicting discoveries regarding the stability of stilbenes has raised questions on the future use of stilbenes [11,12,13]. According to Piñeiro et al. [11], the utilization of the beneficial effects of resveratrol is limited because it is an easily oxidizable and extremely photosensitive compound. In contrast, Prokop et al. [12] showed that piceid and its aglycone resveratrol are stable polyphenols. In solution, *trans*-resveratrol is converted to *cis*-resveratrol when exposed to daylight [14]. The same photoisomerization reaction takes place when the solution is exposed to UV light [15]. According to the authors *trans*-resveratrol was fully isomerized to *cis*-resveratrol when exposed to UV light at 366 nm. Montsko et al. [16] used HPLC-MS and -MS/MS together with UV spectroscopy and semiempirical quantum chemical calculations to examine the UV isomerization reaction of *trans*-resveratrol in solution under 365 nm UV light irradiation. Besides the formation of *cis*-resveratrol, they claimed the formation of an oxidized derivative of *trans*-resveratrol with a triple bond at the centre of the molecule or the formation of a phenanthrene structure. The formation of a trihydroxy phenanthrene was supported also by Tříska et al. [17]. Yang et al. [18] showed that the UV irradiation of *trans*-resveratrol leads to the formation of a highly fluorescent compound, resveratrone, which was properly characterized by UV, MS and NMR. Computational exploration by Rodríguez et al. [19] uncovered the feasibility of singlet oxygen addition to *cis* or *trans*-resveratrol to furnish a dioxetane intermediate which spontaneously cycloreverts to benzaldehydes. Recently, Francioso et al. [20] showed that upon UV-exposure resveratrol in red wine can isomerise from the *trans* to the *cis*-isomer, which may further cyclise to 2,4,6-trihydroxy-phenanthrene. The identifications of possible new compounds derived from *trans* stilbenes have been mainly tentative and based only on UV or MS spectra without utilizing more detailed structural spectroscopy such as NMR.

In summary, the findings about the chemical stability of *trans*-resveratrol and *trans*-piceid in solution are not fully in agreement with each other. Although the *cis*-isomer was formed in every case when exposed to UV or fluorescent light, the formation of additional oxidized product(s) was not observed by all investigators. A clear conclusion about the stability of *trans*-piceid and *trans*-resveratrol in solution cannot be made.

The stability studies of stilbenes have given contradictory results and been concentrated mainly on resveratrol and its glucoside piceid. Thus, the objective of this work was to study the stability of other biologically interesting and readily available stilbenes such as astringin and isorhapontin and their aglucones piceatannol and isorhapontigenin, which have not been studied in detail previously. To resolve this issue, we assessed the effects of light and storage on the stability of *trans* stilbenes and put more effort on the identification and characterization of new compounds formed during our experiments. The isolation and analysis of the compounds derived from the *trans* stilbenes studied were carried out by chromatographic (HPLC, GC) and spectroscopic techniques (UV, MS, NMR).

## 2. Results and Discussion

### 2.1. The Stability of Stilbenes in Solution

The stability of *trans* isomers of the isolated isorhapontin, astringin, piceid, piceatannol, resveratrol and isorhapontigenin in methanol as well as that of crude spruce bark extract dissolved in methanol was monitored for 2 weeks under two different conditions: (1) *light protected*: solutions of isolated stilbenes were stored in a freezer (−20 °C) with protection from light and (2) *light unprotected*: solutions of isolated stilbenes were placed in a laboratory hood and exposed to continuous fluorescent light. Similar conditions were applied to monitor the stability of stilbene constituents in crude extract prior to isolation. *Trans* isomers of all the isolated stilbenes were stable for the duration of the test (2 weeks, 366 h) at −20 °C if protected from light. The recovery [21] of *trans* isomers of five isolated stilbenes (astringin, isorhapontin, piceid, resveratrol and iso-rhapontigenin) varied in the range of 97–101% and that of *trans*-piceatannol 73–106%. These compounds as well as the crude extract were stable for at least two weeks in darkness at −20 °C. Isolated stilbenes were unstable when exposed to fluorescent light. HPLC–UV analysis of *trans*-resveratrol revealed that irradiation with fluorescent light reduced the *trans*-resveratrol peak and a new one appeared with a longer retention time [21].

HPLC-DAD, HPLC-DAD/MS and HPLC-DAD/MS/MS analyses were used for the tentative identification of the new peaks from photoisomerized isorhapontin, astringin, piceid, piceatannol, resveratrol and isorhapontigenin. UV absorption maxima of the new products at ~220 and ~280 nm are characteristic for the *cis*-stilbene chromophore [22]. The determined *m/z* of [M − H]^−^ ion indicated molecular weights similar to the starting material. NMR analysis confirmed that *trans*-stilbenes (isorhapontin, isorhapontigenin, piceid and resveratrol) gave new products as *cis* isomers (Table 1). Light exposure has been shown to cause the isomerization of *trans*-piceid and *trans*-resveratrol to *cis*-piceid and *cis*-resveratrol respectively [15,23] which is in line with our observations.

*trans*-Astringin exposed to fluorescent light suffers a loss of two hydrogen atoms from the *trans*-astringin molecule yielding a new product (Table 1) with maximum absorption at ~260 nm and molecular mass 404 Da as can be deduced from [M − H]^−^ = 403 *m/z*.

**Figure 1:**
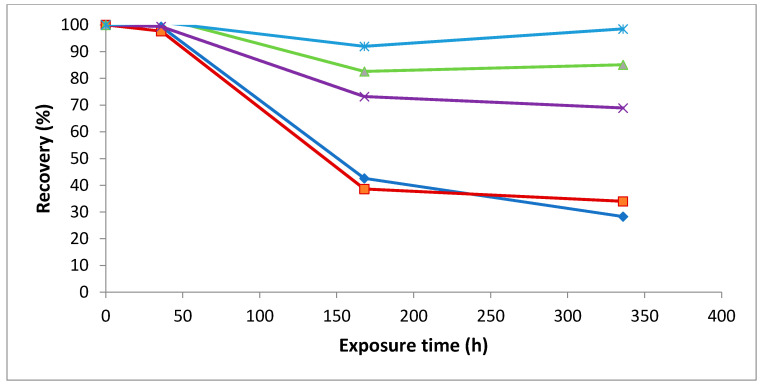
Effect of fluorescent light exposure on the stability of *trans*-stilbenes isolated in MeOH solution. 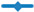 *trans*-astringin, 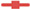 *trans*-piceid, 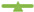 *trans*-isorhapontin, 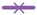 *trans*-resveratrol and 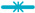 *trans*-isorhapontigenin.

*Trans*-piceid, *trans*-resveratrol and *trans*-astringin showed the highest instability under fluorescent light exposure whereas *trans*-isorhapontigenin and *trans*-isorhapontin were more stable (Figure 1). The steric hindrance due to the methoxy substituent in the structure of isorhapontin and its aglucone discourages the *trans* to *cis* isomerization.

**Figure 2:**
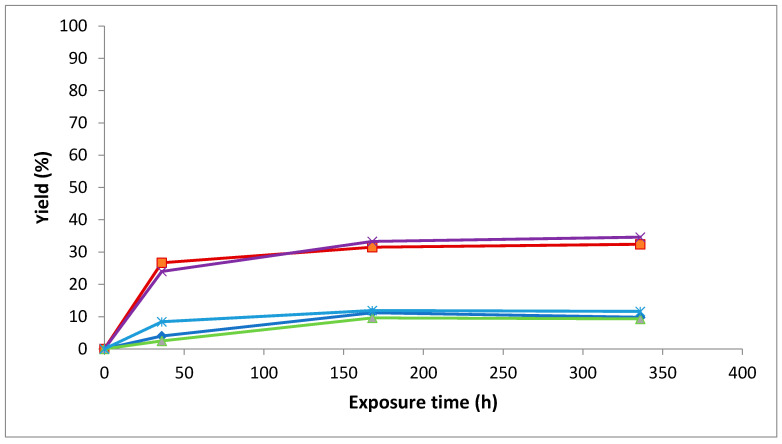
The yield of *cis* stilbenes and a new compound (derived from *trans*-astringin) formed by the exposure of corresponding *trans* stilbenes to fluorescent light in MeOH solution. 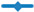 peak derived from *cis*-astringin, 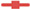 *cis*-piceid, 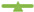 *cis*-isorhapontin, 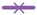 *cis*-resveratrol and 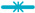 *cis*-isorhapontigenin.

The effect of fluorescent light on the stability of isolated stilbenes can also be seen from the yield of new products (Figure 2). Maximum conversion resulted from the isomerization of the *trans* forms of resveratrol and piceid to their corresponding *cis* isomers after 2 week fluorescent light exposure at room temperature. As shown in Figure 2, the conversion rate is faster for the initial 36 h and becomes slower thereafter.

Crude spruce bark extract solutions were not significantly destablized by exposure to fluorescent light. It is known that in addition to stilbenes, spruce bark contains phenolic compounds such as lignans, flavonoids and tannins [1,7,10] which may influence the action of light on the stilbenes.

### 2.2. Stability Assessment of Stilbenes in Solid Crude Extract

Stilbene glucosides (*trans*-astringin, *trans*-isorhapontin and *tran*s-piceid) in the solid crude extract showed 100% recovery when assessed for their stability under three different conditions: *(1) Light protected:* solid extract in a capped glass vial stored in freezer (−20 °C) with protection from light; *(2) Light unprotected capped glass vial:* solid extract in a capped glass vial exposed to continuous fluorescent light and *(3) Light unprotected uncapped glass vial:* solid extract in glass vial left uncapped and placed on laboratory cabinet to expose it to continuous fluorescent light [21]. Even if in contact with air in uncapped vials, the oxidation or isomerization of the evaluated stilbenes were not observed. In other studies, solid resveratrol and piceid in glass vials protected from light were stable [12], and furthermore the solid *trans*-resveratrol and *trans*-piceid are not very sensitive to UV/fluorescent light, elevated temperature or humidity or atmospheric oxidants at ambient conditions [21,23].

### 2.3. UV Stability of Stilbenes

**Figure 3:**
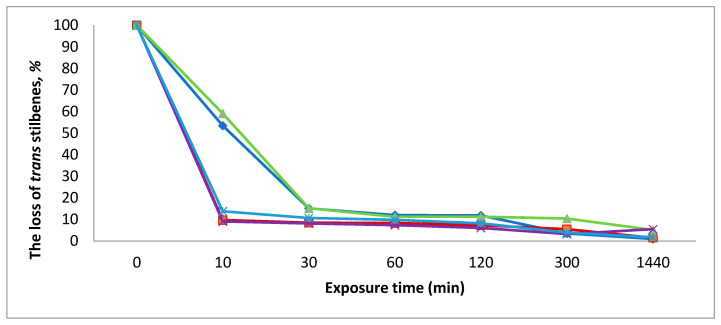
Effect of UV light exposure on the stability of isolated *trans*-stilbenes in MeOH solution. 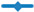 *trans*-astringin, 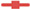 *trans*-piceid, 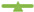 *trans*-isorhapontin, 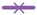 *trans*-resveratrol and 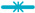 *trans*-isorhapontigenin.

The isomerization of crude *trans* stilbenes extract, hydrolysed (containing stilbene aglucones) and non-hydrolysed (containing stilbene glucosides), to thessss *cis* forms started immediately after exposure to UV light (366 nm) and most of the *trans* structures had disappeared after 10–30 min (Figure 3). *trans*-Astringin and *trans*-isorhapontin showed the lowest rate of disappearance of the *trans* form.

**Figure 4:**
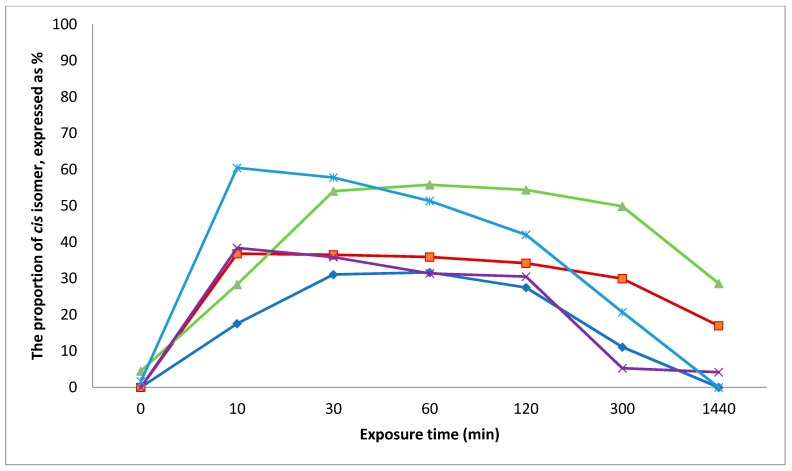
The proportion of *cis* stilbenes due to the exposure of *trans* isomers to UV light in MeOH solution. 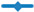 *cis*-astringin, 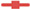 *cis*-piceid, 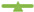 *cis*-isorhapontin, 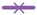 *cis*-resveratrol and 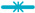 *cis*-isorhapontigenin.

The exposure of the isolated stilbenes in methanol solution, hydrolysed (containing stilbene aglucones) and non-hydrolysed (containing stilbene glucosides), crude extract under UV irradiation lamp (366 nm) caused the isomerization of *trans* stilbenes to *cis* forms. After 10–30 min of UV exposure most of the *trans* structures were absent (Figure 3). The proportion of *cis* stilbenes increased 10–60 min after starting the exposure (Figure 4). After that the proportion of *cis* structures decreased due to transformation of *cis* stilbenes to other products.

The GC-MS information of the *trans* stilbenes isolated and the compounds derived from those during UV irradiation (366 nm) is presented in Table 2. In general, *trans*-to-*cis*-isomerisation took place rapidly and after that all the compounds lost two hydrogens from their structures presumably by oxidation. In the case of piceatannol, the *cis* form was not detected by GC-MS.

In the hydrolysed crude extract, all three *trans* stilbene aglucones isomerized to the *cis* forms, which were the major compounds at 2 h. The new peaks with loss of two hydrogens derived from stilbene aglucones were found from the hydrolysed crude extract after 24 h UV irradiation by GC-MS as in the case of isolated stilbene aglucones. The same was true for the non-hydrolysed extracts containing the *trans* stilbene glucosides.

### 2.4. Isolation and Identification of the New Compounds Formed during UV Irradiation

**Figure 5:**
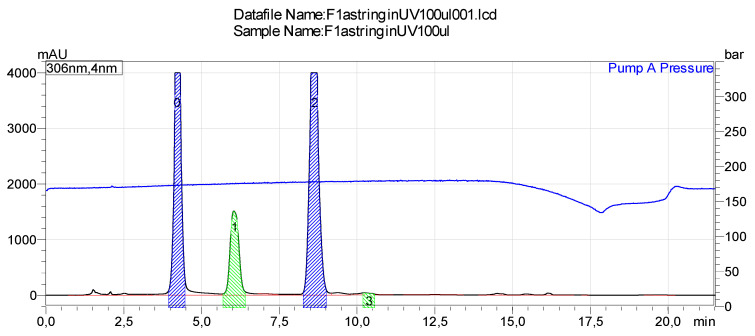
Sample chromatogram of preparative HPLC fractionation of stilbenes derived from UV-exposed astringin.

**Figure 6:**
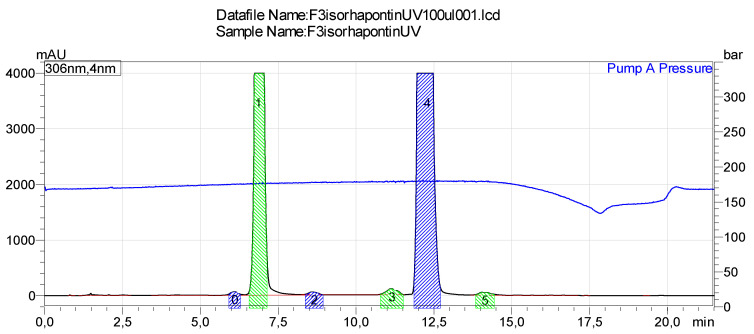
Sample chromatogram of preparative HPLC fractionation of stilbenes derived from UV-exposed isorhapontin.

The new compounds derived from the *trans* stilbenes during UV light exposure were fractionated by using preparative HPLC and identified by NMR and MS. The stilbenes showed isomerization from *trans* to *cis* form during UV light irradiation followed by the loss of two hydrogens (−2H) after a longer time of irradiation. ESI-TOF-MS was used to identify the fractions derived from stilbene glucosides, astringin (Figure 5) and isorhapontin (Figure 6) (Table 3) and EI-MS to identify the fractions derived from hydrolyzed stilbenes piceatannol and isorhapontigenin.

### 2.5. The Structural Elucidation of New Peaks Formed from Astringin (and Its Aglycone Piceatannol) and Isorhapontin (and Its Aglycone Isorhapontigenin)

**Figure 7:**
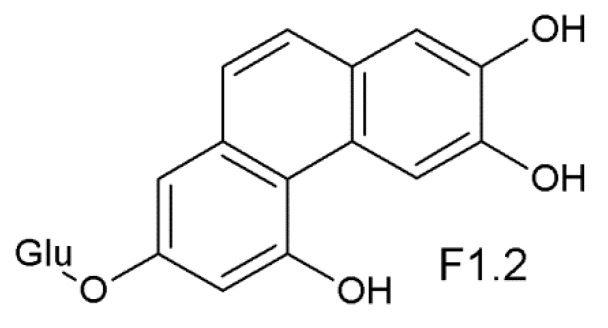
The proposed structure of F1.2, a phenanthrene molecule derived from the stilbene astringin by a photochemical cyclization and dehydrogenation induced by UV irradiation.

For the new compound derived from *trans*-astringin (F1.2), the ESI-MS spectrum showed 403.1036 *m*/*z* as the parent peak. Its molecular mass is two units less than that of *trans*- and *cis*-astringin. In the ^1^H-NMR spectrum, there are six proton signals between 6.0–10.0 ppm. Alkene H signals with a larger coupling constant (ca 12–16 Hz) were absent from the aromatic range. The signal δ 9.008 (1H, s) could be an aldehyde proton, but the aldehyde carbon signal (around 200 ppm) however does not appear in the ^13^C-NMR spectrum. This proton bound to carbon (about 104 ppm) is therefore an aromatic proton. The signals 7.435 (1H, d, *J* = 9 Hz) and 7.319 (1H, d, *J* = 9 Hz) belong to a pair of ortho-coupled aromatic protons. They do not couple with other aromatic protons. The signals 6.968 (1H, d, *J* = 2.4 Hz) and 6.843 (1H, d, *J* = 2.7 Hz) had almost the same coupling constants, so they may be from the same aromatic ring. 2D NMR (COSY, HSQC, and HMBC) also supported these findings. Based on the above evidence the new structure was identified (F1.2) as 7-*O*-β-D-glucosyl-2,3,5-trihydroxyphenanthrene (Figure 7).

**Figure 8:**
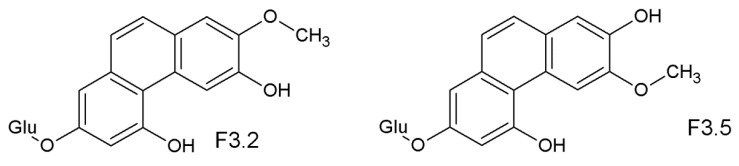
The proposed structures of F3.2 and F3.5, phenanthrene molecules derived from the stilbene isorhapontin due to a photochemical cyclization and dehydrogenation induced by UV irradiation.

For the new compounds derived from *trans*-isorhapontin, the negative ESI-MS spectrum showed the strongest peak at 417.1321 *m*/*z* (for F3.2, F3.3, F3.5) two units less than *trans*- and *cis*-isorhapontin. ^1^H-NMR and 2D NMR signals of F3.2 were almost the same as those in fraction F1.2, except for the OCH_3_ signal at 3.992. F3.5 showed the shift of the methoxy signal to 3.432 ppm, which may be due to the formation of different isomers of phenanthrenes since the positions of hydroxy and methoxy groups adjacent to each other have two alternative arrangements in the *cis* isomer from which is the starting point of phenanthrene formation. F3.3 was the mixture of the fractions F3.2 and F3.5. The new structures identified (F3.2 and F3.5) are 7-*O*-β-D-glucosyl-3,5-dihydroxy-2-methoxyphenanthrene and 7-*O*-β-D-glucosyl-2,5-dihydroxy-3-methoxyphenanthrene (Figure 8).

**Figure 9:**
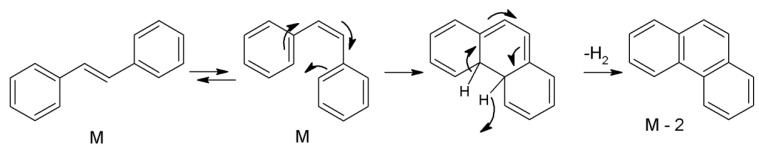
Proposed mechanism of photochemical transformation of *cis* stilbenes to phenanthrenes.

The novel compounds derived from *trans*-piceatannol and *trans*-isorhapontigenin show similar phenanthrene NMR signals. Our NMR data in combination with our UV and MS data suggest that the photochemical transformation of stilbenes to phenanthrene structures goes by way of an intramolecular cyclisation forming a new C-C bond. The phenanthrene structures observed in our studies are supported by earlier data based on the studies of *trans*-resveratrol and its glucoside, piceid [16,17,18,24] and by the studies of pure stilbene molecule itself [25,26]. Our study of various stilbenes isolated from bark extracts now establishes their general behavior exhibited in the photochemical transformation to phenanthrene structures. The suggested electrocyclic mechanism of photochemical transformation of *cis*-stilbenes to phenanthrenes is shown in Figure 9.

**Figure 10:**
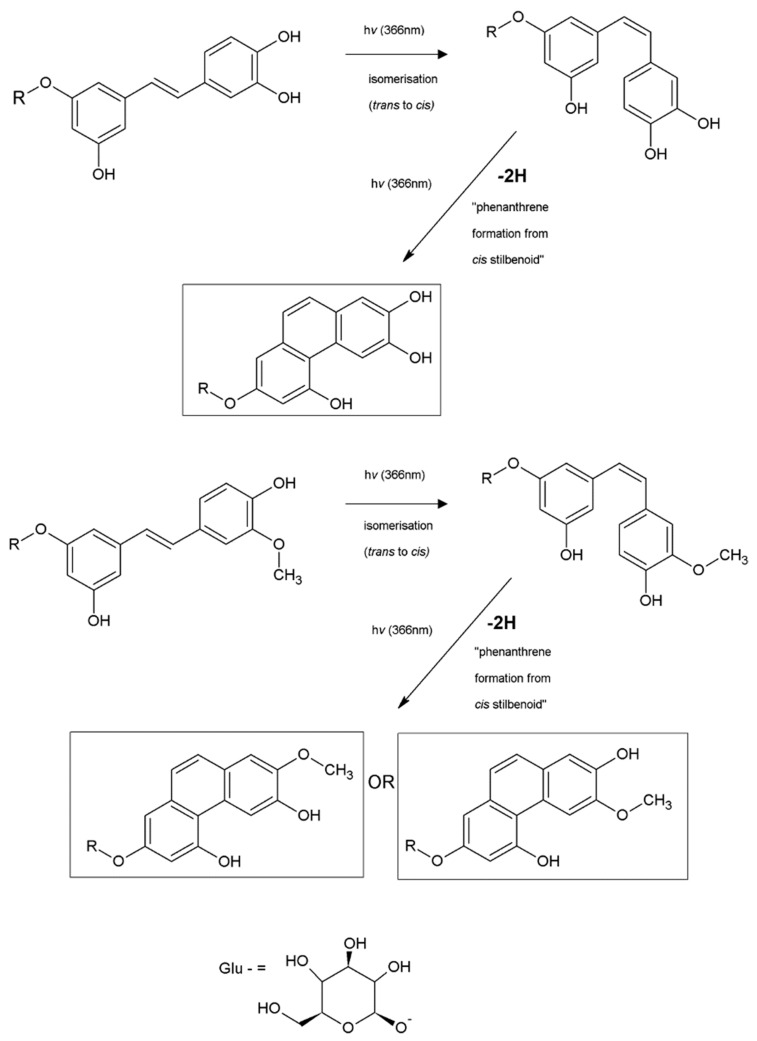
Proposed photochemical transformation of selected *trans* stilbenes (astringin and isorhapontin and their aglycones piceatannol and isorhapontigenin) derived from Norway spruce root bark. R = -H or -*O*-β-D-glucoside (Glu). −2H = loss of two hydrogens

Photochemical transformations of *trans*-astringin and *trans*-isorhapontin (and of their aglycones, *trans*-piceatannol and *trans*-isorhapontigenin) derived from Norway spruce root bark have been reported and described (Figure 10). These molecules formed by photochemical transformation of naturally occurring stilbenes have not been reported earlier in the literature.

The UVA-induced modification of the stilbene-rich inner bark extracts increased the radical scavenging activity [20]. The results also indicate even a slightly increased antimicrobial activity after UVA-modifications [20]. Thus stilbenes have commercial potential as e.g., preservatives.

## 3. Materials and Methods

### 3.1. Bark Material

Thick roots of Norway spruce (*Picea abies* [L.] Karst) were collected from trees growing on mineral soil from Parkano (Lapinneva, Western Finland) in June 2010. The detailed sample information is given in Latva-Mäenpää et al. [1]. The samples were collected from the underground parts of the roots close to the stem. After debarking the bark material of the roots was immediately frozen at −20 °C. It was splintered, freeze-dried for 2–3 days, subsequently ground and stored in a freezer (−20 °C) prior to analysis.

### 3.2. Extraction of Bark Material

The bark extract of Norway spruce was obtained by sequential extraction of the powdered bark (2 g) first with hexane to remove the hydrophobic constituents, followed by 95% V/V ethanol to collect the hydrophilic stilbene constituents using an Accelerated Solvent Extraction (ASE 350, Dionex, Sunnyvale, CA, USA). The ASE sequential extraction method [1,7,21] was: (1) *n*-hexane extraction: temperature, 90 °C; static time, 5 min; two cycles and (2) ethanol:water (95:5 V/V) extraction: temperature, 100 °C; static time, 5 min; two cycles for 2 g sample or three cycles for 3 g of sample. The crude extract was diluted 1:10 with MeOH before injecting 5 µL into the HPLC-DAD, HPLC-DAD/ESI-MS and HPLC-DAD/ESI-MS/MS instruments.

The stilbene content of the non-hydrolysed samples was enriched in an XAD-7HP polymer resin filled column, which was previously conditioned with water. Polyphenolic compounds were absorbed in a resin material and water-soluble compounds such as sugars were washed away with water. Stilbene glucosides were collected from ethanol-water eluates. All samples were stored at −20 °C with light protection until further processing. For producing and isolating hydrolysed stilbene aglucones the other 95% V/V ethanol extract was evaporated to dryness by rotary evaporation at 40 °C and dissolved in 15 mL of sodium acetate buffer (0.1 M, pH 5) solution. 15 mL of β-glucosidase enzyme (3 mg/mL) was suspended in the above solution and incubated in a laboratory oven at 39 °C for 3 days. The hydrolysed samples were enriched in a polymer resin as mentioned above and collected from ethyl acetate eluates. The stilbene glucoside and the stilbene aglucone fract e ions were evaporated to dryness by rotary evaporation at 40 °C and used for preparativHPLC, HPLC, HPLC-DAD/MS and HPLC-NMR analyses.

### 3.3. HPLC-DAD Analyses

Separation of stilbenes from the extract of the root bark of Norway spruce was carried out by HPLC. A Model 1100 series liquid chromatography system (Hewlett-Packard, Santa Clara, CA, USA) with a quaternary pump, degasser, autosampler, column oven and an Agilent 1100 series diode array detector coupled to a Hewlett-Packard Chem Station was used for analysis. HPLC separation was performed on an Xbridge-C18 column (4.8 × 150 mm, 5 μm, Waters, Milford, MA, USA) using a mobile phase of H_2_O and MeOH with a flow rate of 0.8 mL/min. The mobile phase gradient program was 90:10 (t) 0 min, 75:25 (t) 3 min, 60:40 (t) 21 min, 50:50 (t) 30 min, 30:70 (t) 33 min, 10:90(t) 35–36 min and 90:10 (t) 37–39 min. The UV profiles of eluates were monitored with DAD in the range of 200–400 nm. Detection was carried out at 286 nm and 306 nm for *cis* and *trans* stilbenes, respectively (260 nm was also used for detection in UV experiments). Measurements were carried out in duplicate or in triplicate. The mobile phase (H_2_O and MeOH) gradient program was further modified for the structural characterisation of the compounds formed after UV irradiation and this separation was performed by Prominence HPLC instrument (Shimadzu, Canby, OR, USA); the modified mobile phase gradient program was 72:28 (t) 0 min, 60:40 (t) 9 min, 50:50 (t) 13.5 min, 30:70 (t) 15 min, 10:90 (t) 17.5 min and 72:28 (t) 18–21.5 min with a flow rate of 1.4 mL/min.

### 3.4. Isolation of Stilbenes by Preparative HPLC

Preparative isolation was performed using a Waters 600 HPLC instrument on a Waters Xbridge-C18 column (19 × 150 mm, 5 μm) using a mobile phase of water and acetonitrile with a flow rate of 7 mL/min. The mobile phase gradient programs for the non-hydrolysed and hydrolyseSunnd samples were 90:10 (t) 0 min, 85:15 (t) 5 min, 70:30 (t) 25 min, 50:50 (t) 30 min, 10:90 (t) 35 min, 90:10 (t) 37–40 min and 90:10 (t) 0 min, 85:15 (t) 10 min, 70:30 (t) 35 min, 50:50 (t) 38 min, 10:90 (t) 40 min, 90:10 (t) 43–45 min, respectively. The fractions of the hydrolysed extract and the non-hydrolysed extract were collected at 7.5, 11.2, 12.2 min and 13.3, 18.3, 19.5 min. All collected fractions (7 mL each) were evaporated to dryness by a Biotage SB4 evaporator (Uppsala, Sweden) and dissolved in 10 mL of MeOH. The dissolved stilbenes were filtered through a 0.45 μm microporous membrane and stored in freezer at −20 °C until analysis. The chemical structures of the stilbenes isolated were confirmed by NMR and MS.^8^ The mobile phase (H_2_O and MeOH) gradient program was further modified for the isolation of stilbenes for the structural characterization of the compounds formed after UV irradiation and this fractionation was performed by Shimadzu Prominence HPLC instrument; the modified mobile phase gradient program was 72:28 (t) 0 min, 60:40 (t) 9 min, 50:50 (t) 13.5 min, 30:70 (t) 15 min, 10:90 (t) 17.5 min and 72:28 (t) 18–21.5 min with a flow rate of 14 mL/min.

### 3.5. HPLC-DAD/ESI-MS and HPLC-DAD/ESI-MS/MS Analyses

For the online HPLC-DAD/ESI-MS and HPLC-DAD/ESI-MS/MS analyses, an Agilent 1100 Series liquid chromatography system chromatograph coupled to a Bruker Esquire 3000plus ion trap mass spectrometer (Bruker Daltonics, Bremen, Germany) was utilised. The negative ion HPLC-DAD/ESI-MS experiments were conducted using conditions as follows: scan range *m*/*z* = 100–1000; dry temperature, 300 °C; drying gas, 8.0 L/min; nebulizer, 20 psi; capillary potential, 4 kV; spectra averages, 7; rolling averages 1. The HPLC conditions were the same as the one used for HPLC-DAD analyses. HPLC-DAD/ESI-MS/MS of stilbenes was studied by applying a 5 V supplementary radiofrequency (RF) voltage to the end-caps of the ion trap in order to induce collision with the helium buffer gas present in the ion trap.

### 3.6. Stability Assessment of Stilbenes in a Solution and Solid Form

Stock solutions of reference *trans*-resveratrol (290 μg/mL), *trans*-piceatannol (380 μg/mL) and *trans*-piceid (270 μg/mL) were prepared in MeOH. In addition, stock solutions of isolated stilbenes from our study were prepared in MeOH. The stock solutions of isolated stilbenes were *trans*-astringin (120 μg/mL), *trans*-piceid (40 μg/mL), *trans*-isorhapontin (320 μg/mL), *trans*-piceatannol (90 μg/mL), *trans*-resveratrol (14 μg/mL) and *trans*-isorhapontigenin (190 μg/mL).

A 5 mL crude bark extract prior to concentration on a column packed with XAD-7HP ETAX was directly taken for stability study. The stability of the isolated stilbenes, reference stilbenes and crude extract was tested under various conditions described in Table 4. Aliquots of each sample were taken after 0 h (control sample), 24 h, 1 week and 2 weeks for HPLC-DAD, HPLC-DAD/ESI-MS and HPLC-DAD/ESI-MS/MS.

The stability of the solid crude extract was tested under three different conditions (the amounts of dried extracts were in the range of 10–11 mg) described in Table 4. The samples were taken at intervals of 0 h (control sample), 1 week and 2 weeks for HPLC-DAD analysis.

### 3.7. UV Stability of Isolated Stilbenes and Crude Extracts

The UV stability of the isolated stilbenes (in methanol solution), hydrolysed (containing stilbene aglucones) and non-hydrolysed (containing stilbene glucosides) crude extracts under UV radiation were tested by UV lamp with 366 nm wavelength (UV-A radiation). The stilbenes were placed in vials on the work surface of the UV lamp system and samples were taken at intervals of 0 h (control sample), 10 min, 30 min, 1h, 2 h, 5h and 24 h for HPLC-DAD analysis. A GC-MS method [1] was also applied to analyse samples at 0 h, 2 h and 24 h.

Larger amounts of bark-derived stilbenes were isolated by semiprep-HPLC for the structural elucidation of new compounds formed during UV irradiation. The stilbenes isolated were irradiated by UV for 6 days and the compounds formed were isolated by prep-HPLC, dried by vacuum centrifuging and identified by MS and NMR.

### 3.8. Mass Spectrometry (MS) and Nuclear Magnetic Resonance Spectroscopy (NMR)

The mass spectrometry analyses were conducted for stilbene glucosides by high resolution ESI-TOF-MS with negative ion mode and for stilbene aglucones by EI-MS (high resolution sector instrument). Acetonitrile-water was used as the solvent. The NMR analyses were performed by 500 MHz and 300 MHz spectrometers. The ^1^H and ^13^C spectra were recorded in CD_3_OD (300 MHz, 500MHz). ^1^H and ^13^C chemical shifts were referenced to solvent signals of CD_3_OD, δ (^1^H) = 3.31 ppm and δ (^13^C) = 49.86 ppm. The following methods were used to analyse the products formed in UV irradiation studies presented in the chapter concerning the experiments of the UV stability.

For the high-resolution mass spectra (HR-ESI-MS) a JMS-SX102mass spectrometer (JEOL, Tokyo, Japan) was used.

ESI-TOF MS analysis, a Bruker Daltonics micro-TOF-Q I (Bruker Daltonics), the data was processed by means of DataAnalysis 3.2 (Bruker Daltonics). The positive ion ESI-TOF MS analysis was conducted using source conditions as follows: scan range of 0-3000 *m*/*z*; dry temperature, 180 °C: Nitrogen was used as the drying gas, 4.0 L/min; nebuliser, 04. bar. Voltages used were: nebulizer end plate 500V, capillary exit 100 V and capillary potential 4.5 kV.

NMR analysis: 300 MHz Mercury NMR spectrometer (Varian, Palo Alto, CA, USA) and Avance III 500 NMR spectrometer (Bruker Corporation, Billerica, MA, USA) controlled with TopSpin 3.1 (Bruker, Karlsruhe, Germany). All NMR spectra were acquired at 300 K (for the NMR data see the Supplementary Materials).

## 4. Conclusions

*trans*-Stilbenes derived from the bark of Norway spruce are stable compounds when stored carefully and protected from light. A *trans- to cis*-isomerization takes place rapidly upon UV irradiation, but also under fluorescent light after a longer time.

In this study, we identified new compounds derived from stilbenes from the bark of Norway spruce roots. The individual stilbene molecules were isolated from the extracts and their photochemical stabilities (under fluorescent and UV light) were studied. Isomerisation was much faster in the presence of UV light. The molecular structures of stilbenes change by intramolecular cyclisation (new C-C bond and loss of two hydrogens) to phenanthrene structures. This is the first time that these new phenanthrene structures are reported to be derived from stilbenes of spruce bark.

## Figures and Tables

**Table 1:** HPLC-DAD-MS characteristics (t*_R_* (min), UV profile and [M − H]^−^, *m/z*) of the new products after light exposure of stilbene solutions.

Sample Name	Products	t*_R_*(min)	UV Profile	[M − H]^−^, *m/z*
*trans*-astringin	*trans*-astringin	11.8	*trans*-stilbene	405
	new peak	16	λ _max_~260	403
*trans*-piceid	*trans*-piceid	16.3	*trans*-stilbene	389
	new peak	27.5	*cis*-stilbene	389
*trans*-isorhapontin	*trans*-isorhapontin	17.5	*trans*-stilbene	419
	new peak	28.3	*cis*-stilbene	419
*trans*-piceatannol	trans-piceatannol	18.6	*trans*-stilbene	243
	no peak detected			
*trans*-resveratrol	*trans*-resveratrol	25	*trans*-stilbene	227
	new peak	32.3	*cis*-stilbene	227
*trans*-isorhapontigenin	*trans*-isorhapontigenin	26.4	*trans*-stilbene	257
	new peak	33.1	*cis*-stilbene	257

**Table 2:** GC-MS^1^ characteristics of the stilbenes and new products obtained from the UV light (366 nm) exposure experiment.

Sample	Products	Major Compound After UV Irradiation (0 h, 2 h, 24 h)	GC-MS Retention Time (min)	GC-MS (TMSI), *m/z*	HPLC UV Profile
*trans*-astringin	*trans*-astringin	0 h	41.72	532	*trans*-stilbene
	*cis*-astringin	2 h	32.04	532	*cis*-stilbene
	new peak	24 h	34.48	530	λ _max_~260
*trans*-piceid	*trans*-piceid	0 h	38.42	444	*trans*-stilbene
	*cis*-piceid	2 h	30.30	444	*cis*-stilbene
	new peak	24 h	31.77	442	λ _max_~260
*trans*-isorhapontin	*trans*-isorhapontin	0 h	42.45	474	*trans*-stilbene
	*cis*-isorhapontin	2 h/24 h	31.62	474	*cis*-stilbene
	new peak 1	24 h	30.43	472	λ _max_~260
	new peak 2	24 h	34.48	472	λ _max_~260
	new peak 3	24 h	43.54	472	λ _max_~260
*trans*-piceatannol	trans-piceatannol	0 h	22.76	532	*trans*-stilbene
	new peak 1	2 h	21.57	530	λ _max_~260
	new peak 1	24 h	21.57	530	λ _max_~260
*trans*-resveratrol	trans-resveratrol	0 h	20.00	444	*trans*-stilbene
	*cis*-resveratrol	2 h	13.89	444	*cis*-stilbene
	new peak	24 h	18.64	442	λ _max_~260
*trans*-isorhapontigenin	*trans*-isorhapontigenin	0 h	22.55	474	*trans*-stilbene
	*cis*-isorhapontigenin	2 h	15.62	474	*cis*-stilbene
	new peak	24 h	16.36	472	λ _max_~260

**Table 3:** Identification of compounds derived from *trans*-astringin (F1) and *trans*-isorhapontin (F3) by HR ESI-TOF-MS.

Compound	Stilbene	[M-](*m*/*z*)	Calcd.	Formula
**F1.1**	*trans*-astringin	405.1180	405.1186	C_20_H_21_O_9_
**F1.2**	new peak	403.1036	403.1029	C_20_H_19_O_9_
**F1.3**	*cis*-astringin	405.1243	405.1186	C_20_H_21_O_9_
**F3.1**	*trans*-isorhapontin	419.1479	419.1342	C_21_H_23_O_9_
**F3.2**	new peak	417.1321	417.1186	C_21_H_21_O_9_
**F3.3**	new peak	417.1327	417.1186	C_21_H_21_O_9_
**F3.4**	*cis*-isorhapontin	419.1487	419.1342	C_21_H_23_O_9_
**F3.5**	new peak	417.1321	417.1186	C_21_H_21_O_9_

**Table 4:** Stability assessment of stilbenes.

Stability Conditions in Solution Form	Stability CondItions in Solid Form
(1) Light protected: The samples were stored in a freezer (−20 °C) with protection from light in glass vials capped under ambient air conditions	(1) Light protected: The solid extract samples in capped glass vials were stored in a freezer (−20 °C) protected from light.
(2) Light unprotected: The samples in glass vials (capped under ambient air) were kept on laboratory bench, exposed to continuous fluorescent light.	(2) Light unprotected: The solid extract samples in capped glass vials under ambient air conditions were kept on laboratory bench, exposed to continuous fluorescent light.
	(3) Light unprotected: Solid extracts samples in uncapped glass vials were kept on laboratory bench, exposed to continuous fluorescent light and permanent contact with air.

## Supplementary Materials

The following are available on-line. Figure S1: F3.2 fraction: The phenantrene structure formed in the photoisomerization of t*rans*-isorhapotin. ^1^H- and ^13^C-NMR data of F1.2; F1.3; F3.2; F3.4 and F3.5.
