# Supplementary material for: Stability and Photoisomerization of Stilbenes Isolated from the Bark of Norway Spruce Roots
Source: Molecules. 2021 Feb 16;26(4):1036. doi: 10.3390/molecules26041036 (PMC7920084; doi:10.3390/molecules26041036)
Supplement: Supplementary file 1 [file molecules-26-01036-s001.pdf]

Supplementary material

Stability and Photoisomerization of Stilbenes Isolated From the Bark of Norway Spruce Roots

Harri Latva-Mäenpää [1,3], Riziwanguli Wufu [1], Daniel Mulat [1], Tytti Sarjala[2], Pekka Saranpää [2] and Kristiina Wähälä [1,4*]

[1] Department of Chemistry, P.O. Box 55, FI-00014 University of Helsinki, Finland
[2] Natural Resources Institute Finland, Tietotie 2, FI-02150 Espoo, Finland
[3] Foodwest, Kärryväylä 4, FI-60100 Seinäjoki, Finland
[4] Department of Biochemistry and Developmental Biology, P.O. Box 21, FI-00014 University of Helsinki, Finland
* Correspondence: kristiina.wahala@helsinki.fi; Tel.: +358 50 4487502
Tytti Sarjala ORCID 0000-0002-1129-2315
Pekka Saranpää ORCID 0000-0002-1129-2315; Web of Science ResearcherID: C-2114-2008
Kristiina Wähälä ORCID 0000-0003-4082-8622

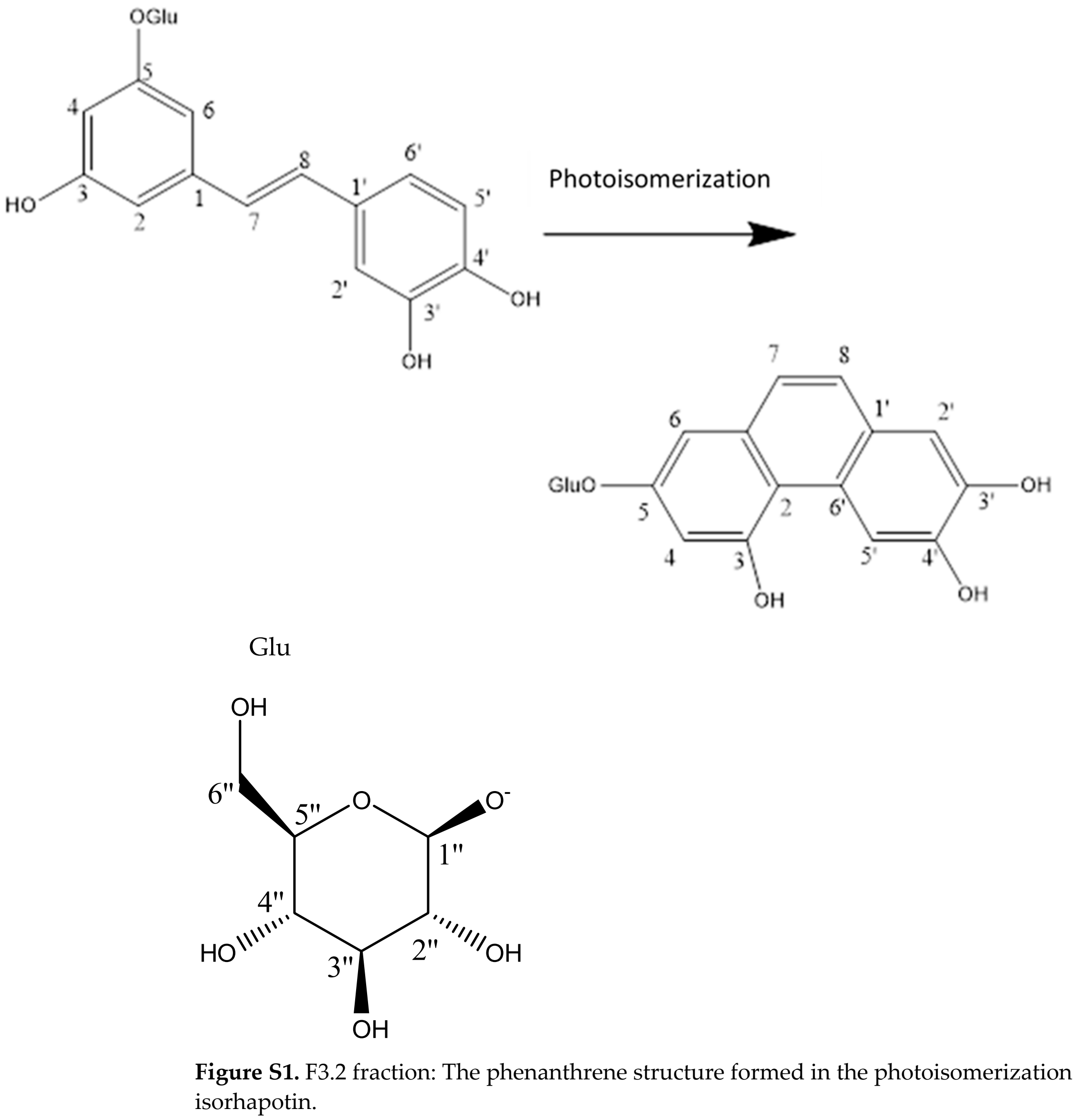

Figure S1. F3.2 fraction: The phenanthrene structure formed in the photoisomerization of *trans* – isorhapotin.

The NMR analyses were performed by Bruker Avance III 500 MHz and Varian Mercury 300 MHz spectrometers. The 1H and 13C spectra were recorded in CD3OD (300

MHz, 500MHz). 1H and 13C chemical shifts were referenced to solvent signals of CD3OD, δ (^{1}H) = 3.31 ppm and δ (^{13}C) = 49.86 ppm.

F1.2 ^{1}H-NMR (300 MHz, CD_3OD): 9,01 (H-5′, 1H, s), 7.44 (H-8, 1H, d, J= 9Hz), 7.32 (H-7, 1H, d, J= 9Hz), 7.13 (H-2′, 1H, s), 6.97 (H-6, 1H, d, J= 2.5 Hz), 6.84 (H-4, 1H, d, J=2.5 Hz), 5.26 (1 H, d, J=7.8 Hz); ^{13}C -NMR (500 MHz, CD_3OD) 136.40 (C-1), 128.53 (C-2), 156.04 (C-3), 103.45 (C-4), 158.19 (C-5), 107.31 (C-6), 125.12 (C-7), 128.36 (C-8), 126.06 (C-1′), 116.06 (C-2′), 146.40 (C-3′), 145.62 (C-4′), 113.04 (C-5′), 114.33 (C-6′), 102.72 (C-1″), 75.01 (C-2″), 78.45 (C-3″), 78.72 (C-4″), 71.38 (C-5″), 62.60 (C-6″).

F1.3 ^{1}H-NMR (300 MHz, CD_3OD): 6.75 (H-2′, 1H,d, J= 1.8Hz,), 6.66 (H-5′, 1H, d, J=8.2 HZ), 6.61 (H-6′, 1H, dd, J=8.2, 1.8 Hz), 6.55 (H-2, 1H, br s), 6.44 (H-7, 1H, d, J= 12.4 Hz), 6.39 (H-6, 1H, br s), 6.73 (H-4, 1H, t, J=2.1 Hz), 6.33 (H-8, 1H, d, 12.4 Hz), 4.67 (H-1″, 1H, d, J= 7.7 Hz); ^{13}C -NMR (500 MHz, CD_3OD) 141.80 (C-1), 110.04 (C-2), 160.93 (C-3), 104.96 (C-4), 160.93 (C-5), 112.22 (C-6), 131.42 (C-7), 129.97 (C-8), 132.46 (C-1′), 117.04 (C-2′), 146.72 (C-3′), 147.69 (C-4′), 117.84 (C-5′), 123.06 (C-6′), 103.34 (C-1″), 75.70 (C-2″), 78.57 (C-3″), 71.83 (C-4″), 78.79 (C-5″), 62.99 (C-6″).

F.3.2 ^{1}H-NMR (500 MHz, CD_3OD): 9.17 (H-5′, 1H, s), 7.57 (H-8, 1H, d, J= 8.5 Hz), 7.44 (H-7, 1H, d= 8.5 Hz), 7.28 (H-2′, 1H, s), 7.04 (H-4, 1H, d, J= 2.25 Hz), 6.85 (H-6, 1H, d= 2.25 Hz), 5.04 (H-1″, 1H, d, J= 7.5Hz), 3.99 (OCH_3, 3H, s); ^{13}C -NMR (500 MHz, CD_3OD) 137.11 (C-1), 128.02 (C-2), 157.48 (C-3), 104.70 (C-4), 159.13 (C-5), 106.95 (C-6), 126.46 (C-7), 128.97 (C-8), 129.23 (C-1′), 115.01 (C-2′), 148.92 (C-3′), 147.78 (C-4′), 117.27 (C-5′), 110.10 (C-6′), 103.23 (C-1″), 75.86 (C-2″), 79.00 (C-3″), 79.13 (C-4″), 72.36 (C-5″), 63.51 (C-6″), 56.99 (OCH_3).

F3.4 ^{1}H-NMR (500 MHz, CD_3OD): 6.85 (H-2′, d, J=2.0Hz), 6.72 (H-6′, dd, J= 8.0, 1.5 Hz), 6.68 (H-5′, d, J=8.0Hz), 6.53 (H-4, br, s), 6.48 (H-8, d, J= 12.3 Hz), 6.42 (H-6, br, s), 6.41 (H-2, br, s), 6.39 (H-7, d, J= 12.3 Hz), 4.71 (H-1″, d, J=7.0 Hz), 3.78 & 3.70 (H-6″, dd, J=12.3, 2.25), 3.63 (OCH_3, 3H, s))3.46 (H-4″, m) 3.41-3.37 (H-2″, overlap), 3.35 (H-5″, overlap), 3.25 (H-3″, m); ^{13}C-NMR (500 MHz, CD_3OD) 142.03 (C-1), 104.79 (C-2), 160.25 (C-3), 110.15 (C-4), 161.08 (C-5), 111.94 (C-6), 129.98 (C-7), 131.09 (C-8), 132.37 (C-1′), 114.26 (C-2′), 149.27 (C-3′), 147.90 (C-4′), 116.83 (C-5′), 124.50 (C-6′), 103.28 (C-1″), 75.66 (C-2″), 78.71 (C-3″), 78.78 (C-4″), 71.93 (C-5″), 63.10 (C-6″), 61.34 (OCH_3, 3H, s)).

F3.5 ^{1}H-NMR (500 MHz, CD_3OD): 7.45 (H-6′, 2H, d, J= 8.5Hz), 7.45 (H-8, 2H, d, J= 8.8 Hz), 7.26 (H-7, 1H, d, J=8.8. Hz), 7.13 (H-5′, 1H, d, J=8.5 Hz), 6.86 (H-4, 1H, d, J= 2.4 Hz), 6.83 (H-6, 1H, d, J=2.4 Hz), 5.22 (H-1″, 1H, J=7.5Hz), 3.48 (OCH_3, 3H, s) ; ^{13}C-NMR (500 MHz, CD_3OD) 138.67 (C-1), 113.90 (C-2), 157.66 (C-3), 103.70 (C-4), 159.02 (C-5), 106.64 (C-6), 124.61 (C-7), 129.51 (C-8), 130.0 (C-1′), 125.33 (C-2′), 151.23 (C-3′), 145.38 (C-4′), 117.59 (C-5′), 126.11 (C-6′), 102.39 (C-1″), 75.80 (C-2″), 79.10 (C-3″), 78.51 (C-4″), 72.16 (C-5″), 63.33 (C-6″), 61.34 (OCH_3, 3H, s).
